# NF-κB/RelA signaling in secretoglobin progenitors mediates plasticity and MMP-induced barrier disruption in house dust mite-induced allergic asthma

**DOI:** 10.1152/ajplung.00066.2024

**Published:** 2024-05-07

**Authors:** Melissa E. Skibba, Allan R. Brasier

**Affiliations:** ^1^School of Medicine and Public Health, University of Wisconsin Madison, Madison, Wisconsin, United States; ^2^Institute for Clinical and Translational Research, Madison, Wisconsin, United States

**Keywords:** asthma, epithelial barrier, matrix metalloproteinase (MMP), plasticity, zinc finger E-box binding homeobox 1 (ZEB1)

## Abstract

The mechanisms how aeroallergens induce sensitization are incompletely understood. The house dust mite (HDM) *Dermatophagoides pteronyssius* (Der p) is a ubiquitous aeroallergen that represents a major cause of allergic rhinitis and asthma. Herein, we tested whether HDM-induced aeroallergen exposure sensitivity is caused by the innate-immune response in small airway epithelial cells. HDM exposure is a rapid activator of NF-κB/RelA in the Secretoglobin (Scgb1a1+) lineage associated with upregulation of NF-κB/RelA-dependent markers of epithelial plasticity. To determine the effect of epithelial NF-κB signaling, NF-κB was depleted in a tamoxifen (TMX)-inducible *Scgb1a1*-CreER^TM^ mouse within a CL57B/L6 background. Corn oil or TMX-treated/RelA-depleted [RelA knockdown (KD)] mice were repetitively exposed to airway HDM challenges to induce airway hyperresponsiveness (AHR). Strikingly, we observed that HDM induces hallmarks of epithelial plasticity through upregulation of the mesenchymal core factors SNAI1 and ZEB1 and production of metalloproteinase (MMP)9 that are RelA-dependent. Downstream, HDM-induced mucous metaplasia, Th2 polarization, allergen sensitivity, and airway hyperreactivity were all reduced in the RelA-depleted mice. Mechanistically, HDM-induced functional and structural barrier disruption was dependent on RelA signaling and associated with active MMP secretion into the bronchoalveolar lavage fluid. To establish the role of MMP2/9 in barrier disruption, we observe that a small-molecule MMP inhibitor (SB-3CT) blocked HDM-induced barrier disruption and activation of plasticity in naïve wild-type (WT) mice. Loss of functional barrier was associated with MMP disruption of zona occludens (ZO)-1 containing adherens junctions. Overall, this data indicates that host innate signaling in the Scgb1a1+ progenitors is directly linked to epithelial plasticity, MMP9 secretion, and enhanced barrier permeability that allows allergen penetration, sensitization producing allergic asthma (AA) in vivo. We propose that maintenance of epithelial integrity may reduce allergic sensitization and AA.

**NEW & NOTEWORTHY** Allergic asthma from house dust mite (HDM) allergy causes substantial morbidity. This study examines the dynamic changes in small airway epithelial cells in a mouse model of HDM exposure. Our findings indicate that NF-κB/RelA signaling mediates matrix metalloproteinase production, disrupting the epithelial barrier resulting in allergic sensitization. Our findings bring new insight into mechanisms for epithelial cell-state change in the allergen response, creating a potential therapeutic pathway for maintaining barrier function in asthma.

## Graphical abstract

**Figure:**
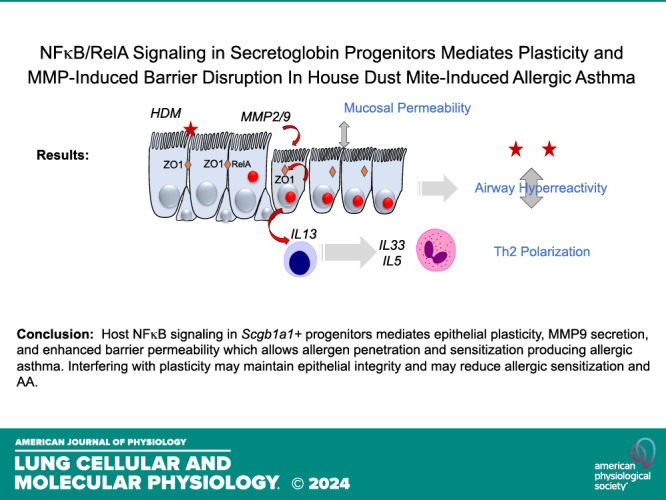

## INTRODUCTION

Asthma is a chronic and heterogeneous disease associated with mucosal inflammation and recurrent episodes of reversible airway obstruction (1). The most common phenotype, allergic asthma (AA), is characterized by aeroallergen sensitization, Th2 polarized inflammation with eosinophilia, airway hyperresponsiveness (AHR), and airway remodeling (1, 2). Worldwide, some 300 million (M) individuals suffer from AA (3), and 600 M people suffer from upper airway mucosal allergy, allergic rhinitis (4). One of the most common allergies is to *Dermatophagoides pteronyssinus*, the house dust mite (HDM). HDM allergen is detected in >80% of US households (5); moreover, 40%–85% of patients with AA are sensitized to this spp. (6) with 80–90% of circulating IgEs are directed to this antigen (7). Although we know that the presence of aeroallergen sensitization increases the risk of AA and allergic rhinitis, the mechanisms how aeroallergens produce sensitization are incompletely understood. Despite its ubiquitous exposures, and ability to activate the innate response, not all individuals who are exposed to HDM develop sensitization and AA. Improved mechanistic understanding of this process will substantially impact health care utilization, absenteeism, and quality of life caused by mucosal allergies (8).

As the first physical barrier encountered by aeroallergens, the airway epithelium plays an important role in initiating allergic sensitization (9). Under normal conditions, the epithelial barrier is produced by cell-cell adhesion complexes (tight junctions) formed between apical surfaces that limit macromolecular transport (10). With barrier disruption, aeroallergens can penetrate into the interstitium stimulating adaptive immunity. Although barrier disruption is characteristic of established AA (11), the role of barrier disruption in the initial stages of sensitization is not well understood.

In naïve (nonsensitized) individuals, aeroallergens are plant- and animal-derived products that activate the epithelium, triggering inflammation and injury/repair processes downstream of pattern recognition receptors (PRRs) (12, 13). For example, the most extensively studied common aeroallergens, such as ragweed, cat-dander, and house-dust mite (HDM) activate distinct PRRs, including Toll-like receptor (TLR)4 and protease-activated receptor (PAR)2 that converge on the NF-κB innate signaling pathway (14–17). NF-κB is an inducible transcription factor that integrates diverse PRR signaling pathways into Th2 cytokine expression. Upon activation, NF-κB complexes translocate into the nucleus to activate proinflammatory chemokines (thymic stromal lymphopoietin, IL-33, and IL-25) and cellular plasticity gene networks by regulated transcriptional elongation (18, 19). This innate mucosal immune response triggers complex, multicellular interactions between recruited neutrophils, natural killer (NK), and dendritic cells leading to Th2 polarized immunity (9). Although its role in innate inflammation is well-established, the role of NF-κB signaling in epithelial injury/repair is not well understood.

The airway epithelium is composed of regionally distinct epithelial cell types that play distinct roles in ciliary clearance, mucous production, innate immunity, and injury/repair (20–22). Recently, attention has been directed to the unique function of specialized Secretoglobin (*Scgb1a1*)-expressing epithelial cells (Club and Goblet progenitors) as critical mediators of airway inflammation and regeneration. Cell lineage studies have shown that *Scgb1a1*^+^ population serve as innate sensors to fungal and animal (cat dander) allergens, playing central roles in neutrophilic inflammation and allergic sensitization (16, 23). In addition to their role in mucosal inflammation, *Scgb1a1-*expressing progenitors play key roles in epithelial repopulation after injury. In particular, genetic knockouts of innate signaling in this *Scgb1a1*^+^ population enhances airway plasticity through mucosal transforming growth factor (TGF)β secretion (16, 24, 25), a major signature of injury/repair pathways (22). As normal precursor cells that differentiate into ciliated and mucus-secreting cells, including Club and goblet cells, *Scgb1a1* expressing basal cell progenitors de-differentiate, migrate, and repopulate distal airways and alveolar type 1 cells (22, 26, 27). This process of de-differentiation is referred to as epithelial “plasticity” as these cells are in a meta-stable state, expressing characteristic signatures of epithelial-mesenchymal transition (EMT) and yet still retaining largely epithelial characteristics (28–30). This phenotypic change permits de-differentiated cells to migrate to sites of injury, and as a reversible cell-state change, re-differentiate into specialized epithelium (18, 28, 30, 31).

In this study, we examine how innate signaling-induced epithelial plasticity is related to AA in response to HDM allergen (32). Although previous work has shown that HDM induces inflammation and airway hyperreactivity via the noncanonical NF-κB pathway (17), we instead observe that HDM acutely activates RelA signaling, the *sine qua non* of the canonical pathway, and induces epithelial plasticity. To determine the role of RelA in the *Scgb1a1^+^* population, we depleted RelA by inducible Cre-Lox system and subjected the RelA knockdown (KD) mice to repetitive HDM exposure. We observe that epithelial plasticity and functional and physical disruption of the epithelial permeability barrier were blocked by RelA depletion, indicating the host innate response signaling actively participates in epithelial barrier disruption. Noting that metalloproteinase (MMP) expression is a major target of NF-κB-mediated plasticity programs, we examined their role using a potent MMP2/9 inhibitor. SB-3CT blocked HDM-induced MMP2/9 activities in the bronchoalveolar lavage fluid (BALF), normalizing barrier function, reducing epithelial plasticity and preventing cell surface zona occludens (ZO)-1 loss. Furthermore, the HDM-activated Th2 polarization, sensitization, and airway hyperreactivity (AHR) were blocked by RelA KD. Concluding, RelA signaling in the *Scgb1a1*^+^ epithelial population plays a central role in the innate response to HDM, coupled to epithelial plasticity, barrier disruption linked downstream with sensitization, and AHR. These findings extend our understanding of the complex interplay between environment and epithelial plasticity responses in AA.

## MATERIALS AND METHODS

### Animal Study

Animal experiments were performed according to the NIH Guide for Care and Use of Experimental Animals and approved by the University of Wisconsin-Madison Institutional Animal Care and Use Committee (Approval No. M006067).

### Animal Treatment

RelA depletion in the Secretoglobin (*Scgb1a1*)-CreER^TM^ × RelA^fl/fl^ mouse (18) was achieved by tamoxifen (TMX) injection. TMX (Sigma Aldrich, St. Louis, MO; T5648) was dissolved in 10% ethanol and 90% corn oil for a 10 mg/mL working solution and administered at a dose of 1 mg/day via the intraperitoneal (ip) route for 10 days. Control mice were *Scgb1a1-*CreER^TM^ × RelA^fl/fl^ mice treated with equivalent volume of corn oil. Both male and female mice aged 3- to 4-wk old were used. Animals were rested for 3 wk prior to HDM exposure. Thereafter, mice were exposed to HDM (10 µg/kg) via the intranasal (in) route on *days 1*–*5*, *9*–*10*, and 16 before euthanasia, wherein serum and lung tissue were collected for analyses or airway physiology measurements.

For inhibition of MMP2/9, 250 µg of SB-3CT (TCI, OR; B5630) was dissolved in 100 µL of PBS and 10% DMSO and administered daily via the intraperitoneal route along with HDM for 4 days. Control animals received solvent. Animals were euthanized on *day 5*.

### Cell Count and Differentials

To collect bronchoalveolar lavage fluid (BALF) cells, BALF was spun at 1,000 *g*. The supernatant was saved for protein analysis and the cell pellet resuspended in red blood cell lysis buffer (Qiagen, Germantown, MD) centrifuged again at 1,000 *g*. The remaining leukocytes were resuspended in PBS with 3% bovine serum and counted using a TC20 automated cell counter (Bio-Rad, Hercules, CA).

Differential cell counts were performed on cells centrifuged onto Superfrost Plus microscope slides (Thermo Fisher Scientific, Waltham, MA) using the StatSpin CytoFuge 2 (HemoCue, Ängelholm, Sweden) according to manufacturer’s recommendations. Slides were fixed in 100% methanol, stained with Wright Giemsa (Thermo Fisher Scientific), immediately photographed, and counted using the Echo Revolve (ECHO, San Diego, CA) at ×60.

### Immunostaining

The right lung was fixed in 10% (vol/vol) neutral buffered formalin for 1 day and processed as described previously (16). Sections were stained with primary anti-ZEB1 antibody (Proteintech, 1:100; 66279-1) or Snail (Proteintech, 1:100; 13099-1-AP), washed, and detected with anti-mouse Alexa Fluor 647 Ab (Thermo Fisher Scientific, 1:1,000; A-31571). For the ZO-1 staining, mouse tissue was flash frozen in a 1:1 solution of optimal cutting temperature (OCT) and PBS using dry ice. Hundred percent (100%) acetone fix was used prior to block with Animal-free Blocking Solution (Thermo Fisher Scientific; NC9592609) and stained with monoclonal ZO-1 conjugated to Alexa-647 (Santa Cruz Biotechnology, 1:200; sc-33725). Both paraffin- and frozen-embedded tissue were mounted in DAPI (ProLong Gold Antifade, Thermo Fisher Scientific; P36935) to visualize nuclei. Antibody specificities were verified by appropriate intracellular staining patterns, absence of signal in RelA-deficient tissues (Bioss, 1:100; bs-0465R), and enhanced signals versus IgG control. Alcian blue was performed using conventional techniques.

### Measurement of Epithelium Permeability

NHS-Biotin was prepared using 125 μg of EZ-Link Sulfo-NHS-Biotin (Thermo Fisher Scientific, Waltham, MA; 21217) in 25 μL of 1 mM CaCl_2_ (MilliporeSigma, Burlington, MA). The animal was lightly sedated 1 h prior to euthanizing and 25 μL of the biotin solution was administered intranasally. Whole lungs were flash frozen in OCT buffer and 8 µm sections were cut. The tissue was stained with Streptavidin-Texas Red (1:200, Millipore Sigma; 189738) and then mounted with a DAPI nuclear stain. Imaging was performed using a Nikon A1RS Confocal Microscope with a ×20 magnification. Quantification and analysis were done by measuring the nuclear intensity of positive cells surrounding the major airways with ImageJ software. Data were expressed as a mean of scores recorded by two blinded investigators.

FITC-Dextran (Thermo Fisher Scientific, 10 mg/kg body wt in PBS; D3305) was administered intranasally into the airways 1 h prior to blood collection via the saphenous vein. Blood was collected in EDTA tubes before centrifugation at 7,000 rpm for 10 min. Fluorescence intensity of FITC-Dextran was determined at an excitation wavelength of 485 nm and an emission wavelength of 528 nm. Values were determined via a standard curve, as recommended by the manufacturer’s instruction.

### Flow Cytometry

The left lung was digested in Dispase II (100 mg/mL), Collagenase A (10 mg/mL), DNAse I (1,500 kU/mL), and CaCl_2_ (0.25 M) into a single cell solution using a 40 μM filter. Prior to fixing the cells in 4% paraformaldehyde, cells were stained with LIVE/DEAD Fixable Violet Dead Cell Stain Kit (Thermo Fisher Scientific; L34987), according to the manufacturer’s instructions. After fixation, the cells were blocked with CD16/32 (1:1,000, Biolegend; 101302). For intracellular stainings, saponin (1 µg/mL, MilliporeSigma) was used in the blocking buffer. Primary anti-CD45 (1:50, Biolegend; 50605954), CD326 (EpCam, 1:100, Biolegend; 50163756), NF-κB/RelA (1:100, Bio-Techn; IC5078G), and CC10 (Uteroglobin, 1:100, Thermo Fisher Scientific; 102469) antibodies (Abs) were used. Cells were washed and resuspended in FACS Flow buffer. Flow cytometry was performed by a Thermo Fisher Attune with Fluorescence Minus One (FMO) standards to verify the antibodies. Histograms were generated and analyzed using FlowJo analysis software (FlowJo, Ashland, OR).

### Quantitative Real-Time PCR

Total RNA from the left lung was extracted and cDNA was prepared using LunaScript RT SuperMix Kit (New England Biolabs, MA). For quantitation of specific transcripts, mouse Taqman or Luna Universal qPCR Master Mix and gene-specific primers (Supplemental Table S1) were used, according to manufacturer’s instructions. Relative changes in gene expression were quantified relative to control GADPH or PPIA transcripts using the ΔΔCt method.

### Zymography

Equal volumes of decellularized BALF were fractionated on a 10% (vol/vol) SDS-polyacrylamide gel copolymerized with 0.1% (wt/vol) gelatin. After electrophoresis, the gel was washed in Wash Buffer (2.5% Triton X-100, 50 mM Tris-HCl, pH 7.5, 5 mM CaCl_2_, and 1 µM ZnCl_2_) twice for 30 min. Then the gel was then incubated in Reaction Buffer (1% Triton X-100, 50 mM Tris-HCl, pH7.5, 5 mM CaCl_2_ and 1 µM ZnCl_2_), at 37°C overnight followed by Coomassie Blue staining. Discolored bands in the gel were visualized to quantify gelatinase activity.

### Airway Physiology Measurements

Airway physiology was measured using flexiVent (SCIREQ, Montreal, QC, Canada) on sedated mice. Briefly, the mouse was mechanically ventilated and administered pancuronium (1 μg/mg/mouse; P1918). Increasing doses of methacholine (MCh, from 1 to 50 mg/mL; A2251) were introduced via nebulization attached to the ventilator circuit. Respiratory system input impedance was derived from data collected using the low-frequency forced oscillation technique (LFOT), and tissue damping (*G*), elastance (Ers), and tissue elastance (Rrs) were determined, by iteratively fitting the constant-phase model to input impedance. Data are reported as an average of all data points. Figure 8 uses data only from 10 mg/mL MCh.

### IgE Assay

Arterial blood was collected in a vacutainer. After clotting, serum was prepared by centrifugation. IgE concentration was measured by ELISA (Novus, Centennial, CO; NBP275005) according to manufacturer’s instruction.

### ELISA

Cell-free BALF (50 µL) was added directly to the Mouse Total MMP-9 Quantikine ELISA Kit (R&D Systems, MN; MMPT90), Mouse IL-6 DuoSet ELISA Kit (R&D Systems; DY406), and Mouse TGF-β1 DuoSet ELISA Kit (R&D Systems; DY1679) along with the Sample Activation Kit 1 (DY010) to detect active TGF-β1. These assays were performed according to the manufacturer’s instructions. BALF was normalized to the total volume of BALF collected.

### Statistical Analysis

Differences among the means were tested for significance by ANOVA with Fisher’s least significance difference test. Individual groups were compared using a post hoc Tukey’s statistic. Significance was reached when the *P* value was 0.05. Values presented are means ± standard deviation (SD).

## RESULTS

### Intranasal HDM Acutely Activates the Canonical NF-κB Pathway

**Figure 1.**
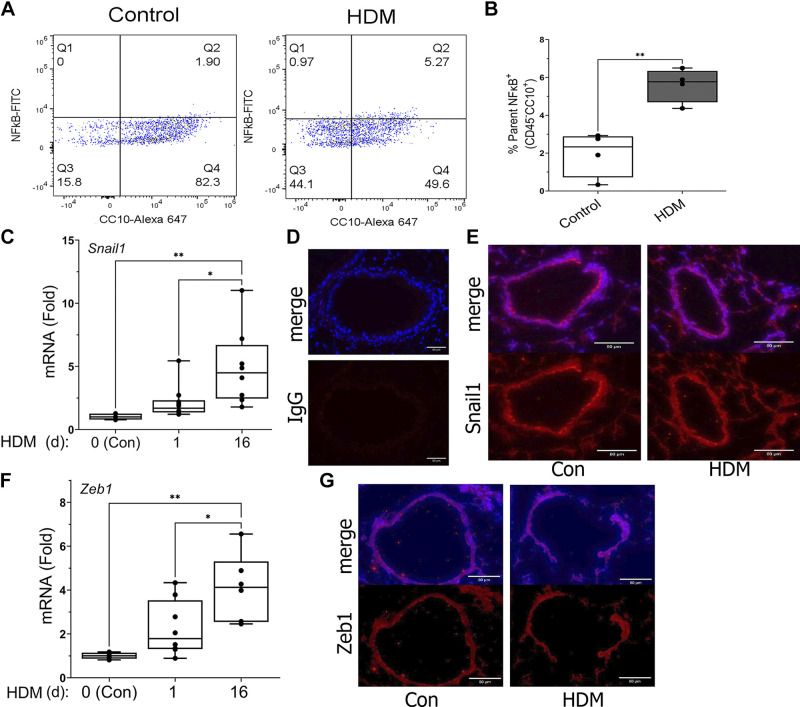
House dust mite (HDM) activates Club cell RelA signaling and epithelial mesenchymal plasticity. *A*: quantitation of RelA in Scgb1a1^+^/CC10 cells. C57BL/6 mice were exposed to a single intranasal administration of HDM (10 μg/mL). Twenty-four hours later, whole lung were dissociated, and stained with anti-CD45, RelA, and EpCam. Cells were gated for CD45−, and live cells were quantified for EpCam (*X* axis) and RelA (*Y* axis). *B*: quantitation of total cellular NF-κB in EpCam+ cells (*n* = 4 animals/treatment condition). *C*: changes in *Snai1* mRNA in total lung RNA were measured after 1 day or 16 days of HDM treatment. For each treatment condition, shown is fold change in *Snail1* mRNA relative to PBS-treated WT, normalized to *Ppia* mRNA (internal control). Symbols are individual animals (*n* = 8 animals/treatment condition). Boxes are 25–75% interquartile range. *D* and *E*: immunofluorescence microscopy (IFM) staining (nuclei are counterstained with DAPI). *D*: shown separately are IgG and merged images. Note the absence of signal in the IgG stained sections. Scale bars are 80 µm. *E*: staining for SNAIL1 in control or HDM (1 day of treatment). *F*: changes in normalized *Zeb1* mRNA as a function of HDM treatment. Data are presented as in (*C*). *G*: IFM for ZEB1 (1 day of treatment). *P < 0.05; **P < 0.01, post hoc Tukey.

To understand the interplay between innate and adaptive immune response involved in allergic sensitization, we investigated host response to HDM airway exposures. Previous work has shown that HDM activates inflammatory cytokines and neutrophilic inflammation after HDM exposure (17). However, because of the diverse cell types capable of responding to HDM allergen in the airway, including resident alveolar macrophages, epithelial cells, and other innate cells, we examined canonical NF-κB pathway activation in *Scgb1a1*^+^ epithelial cells after a single HDM administration using multiparametric flow cytometry. Here, single-cell lung preparations were stained with anti-CD45, RelA, and CC10 antibodies (Abs), and gated on CD45^−^ (Supplemental Fig. S1 and Fig. 1*A*). RelA expression is a characteristic of NF-κB activation in response to diverse conditions (18, 24). We observed that the 1.90% of CC10^+^/CD45^−^ cells stained for RelA in PBS-treated mice that increased to 5.6% (*n* = 4 independent lungs, *P* = 0.0028, two-tailed *P* test, Fig. 1*B*). These data indicate that HDM activates the canonical NF-κB pathway in *Scgb1a1^+^* lung epithelial cells.

Previously, we demonstrated that RelA activation drives the process of epithelial-mesenchymal plasticity important in the mucosal injury response (18, 31). Plasticity is a partial mesenchymal-like cell state indicated by the expression of the mesenchymal transcription factor core complex, snail family transcriptional repressor 1 (SNAI1)/zinc finger E-box binding homeobox 1 (ZEB1). To explore whether a plasticity signature was evoked by HDM, we analyzed *Snai1/Zeb1* expression in wild-type (WT) mice after a single exposure or 16 days of every other day HDM treatment. Interestingly, we observed that HDM induced a significant, time-dependent increase in *Snail1* mRNA from 2.1 ± 1.3-fold [*P* value, not significant (n.s.)] after a single treatment to 4.91 ± 3.0-fold after 16 days of treatment (*P* = 0.0072, Tukey’s post hoc, Fig. 1*C*). Interestingly, SNAIL1 expression was detected in epithelial cells in PBS-treated controls and did not appreciably change in response after 1 day of HDM treatment (Fig. 1*D*).

Similarly, *Zeb1* mRNA increased to 2.3 ± 1.3-fold after 1 day of treatment (*P* = 0.02) to 4.1 ± 1.5-fold after 16 days of HDM treatment (*P* = 0.001, Fig. 1*D*). As with SNAIL1 staining, ZEB1 protein was observed in both control and HDM-stimulated epithelium and was only slightly increased 1.2-fold 1 day after HDM treatment (Fig. 1*E*). These data indicated to us that a single HDM exposure initiates the epithelial plasticity program, but is not sufficient for a full phenotypic change.

### RelA Signaling in *Scgb1a1*^+^ Progenitors Mediates Cellular Inflammation to HDM

**Figure 2.**
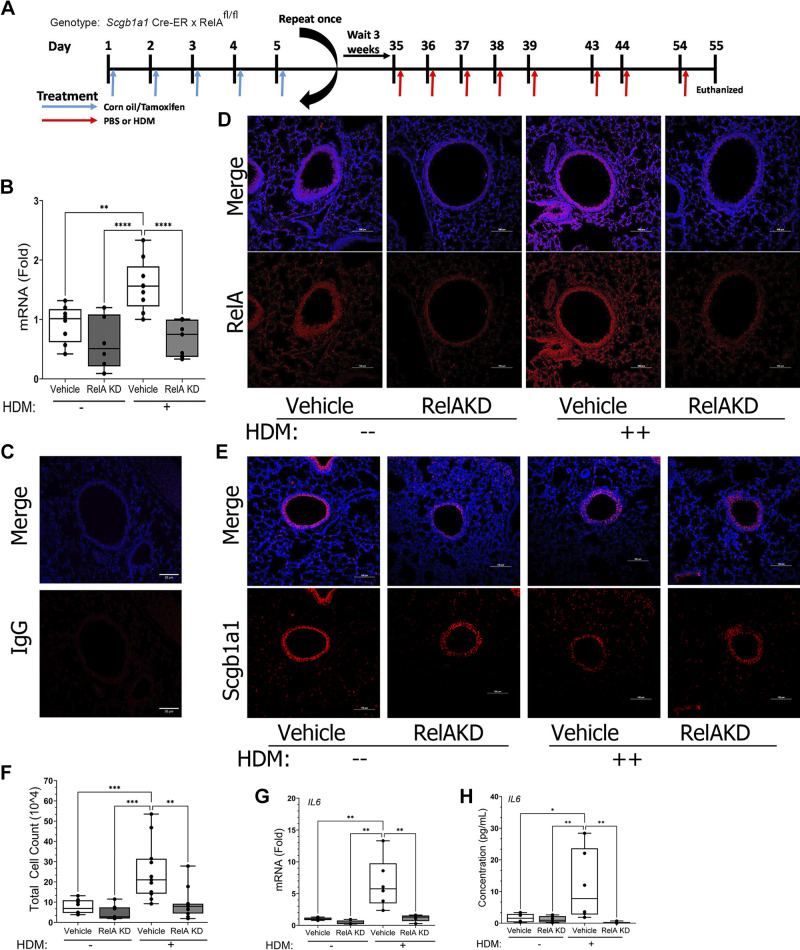
Effects of RelA knockdown (KD) in house dust mite (HDM)-induced allergic asthma (AA) in mice. *A*: experimental strategy for HDM-induced aerosol sensitization and challenge. *Scgb1a1*-CreER^TM^ × RelA^fl/fl^ mice (both sexes) in the C57BL/6J background were administered 10 doses of tamoxifen (TMX) or vehicle (corn oil) via the intraperitoneal (ip) route. Three weeks later, mice (both sexes) were treated with daily challenges of HDM or PBS via the intranasal (in) route. *B*: Q-RT-PCR of *RelA* mRNA. Total lung RNA was harvested and analyzed. Shown is fold change normalized *RelA* mRNA. Symbols are individual animals (*n* = 8 animals/treatment group). Boxes are 25–75% interquartile range. *C–E*: immunofluorescence microscopy (IFM) in vehicle or RelA KD after HDM challenge. *C*: IgG stained controls. Shown separately are IgG and DAPI channels. *D*: effect of HDM and TMX on RelA. Shown separately are RelA stained and merged channels. Note the increase in RelA in vehicle-treated animals after HDM and the loss of RelA after TMX treatment. *E*: Scgb1a1 staining of adjacent sections. Note the loss of RelA in Scgb1a1-expressing epithelium. *F*: total cell count in bronchoalveolar lavage fluid (BALF) expressed as cells × 10^4^/mL. *G*: Q-RT-PCR of NF-κB-dependent *Il6* mRNA expression in whole lung (*n* = 6 mice/treatment). Data are presented as in *B*. *H*: IL6 levels in bronchoalveolar lavage fluid (BALF). IL6 was determined by ELISA and normalized to volume recovered/mouse (*n* = 6 mice/treatment). Note the inhibition of *Il6* expression in the RelA KD mice to control levels. Statistical symbols are: *P < 0.05; **P < 0.01; ***P < 0.005; ****P < 0.0001; post hoc Tukey.

The *Scgb1a1* progenitor cell population has emerged as an important mediator of small airway inflammation in response to diverse PRRs. Using three-dimensional (3-D) clearing and light sheet microscopy, we observed that *Scgb1a1* progenitor cells in the mouse lung are distributed in a punctate pattern scattered throughout small airways and terminal bronchioles into alveoli (16). Our previous standardization of this model showed that TMX administration to *Scgb1a1*-CreER^TM^ × RelA^fl/fl^ mice induces recombination and time-dependent RelA knockdown (KD) in this population (16, 24, 25). TMX- or corn oil-treated mice were subjected to a standardized intranasal challenge of HDM producing allergic sensitization and AHR (Fig. 2*A*). Control mice were *Scgb1a1*-CreER^TM^ × RelA^fl/fl^ mice treated with corn oil.

To confirm that TMX treatment perturbed the RelA pathway, *RelA* mRNA was measured in whole lung extracts by Q-RT-PCR. Relative to PBS-treated controls, HDM induced a 1.6 ± 0.4-fold increase in RelA mRNA in corn oil-treated animals, confirming the HDM-induced upregulation of RelA by flow cytometry (*P* = 0.003, post hoc Tukey, *n* = 6; Fig. 2*B*). By contrast, TMX treatment reduced *RelA* mRNA by 40% in PBS-challenged animals to 0.6 ± 0.4-fold (*n* = 6; *P* < 0.0001, post hoc Tukey, Fig. 2*B*). Furthermore, in response to HDM, TMX treatment reduced *RelA* mRNA by 30% to 0.7 ± 0.4-fold (*P* < 0.0001, post hoc Tukey, *n* = 9; Fig. 2*B*).

To confirm the depletion of RelA in the Scgb1a1+ population described in earlier experiments (24, 33), immunofluorescence microscopy (IFM) was performed. In corn oil-treated controls, relative to IgG control staining (Fig. 2*D*), RelA staining was strongly detected in the small airway epithelium and throughout the alveoli (Fig. 2*D*) that was reduced by TMX treatment alone (Fig. 2*D*). By contrast, a threefold increase in RelA staining was observed in corn oil-treated controls after HDM treatment (Fig. 2*D*) that was inhibited by TMX treatment to levels than the PBS-challenged, corn oil-treated controls (Fig. 2*D*). Notably, the epithelium where TMX reduced the expression of RelA also stained strongly for SCGB1A1 (Fig. 2*E*), indicating tissue specificity for the Cre expression, as previously demonstrated (24, 33).

Cellular inflammation in the bronchoalveolar lavage fluid (BALF) was quantitated in the corn oil- and TMX-treated *Scgb1a1*-CreER^TM^ × RelA^fl/fl^ mice in response to PBS or repetitive HDM exposure. In corn oil-treated mice, HDM induced a fourfold increase in total leukocytes from 4.6 ± 3.3 × 10^4^ to 19 ± 20 × 10^4^ cells/mL (*P* < 0.001, post hoc Tukey, *n* = 6; Fig. 2*F*). The increased leukocytes were primarily in the neutrophil population (Supplemental Fig. S1). In striking contrast, the BALF cell counts in the TMX-treated mice were similar to that of control, PBS-exposed mice (6.1 ± 2.7 × 10^4^ cells/mL, *P* < 0.002 compared with HDM-treated corn oil-treated animals, Fig. 2*F*). These data indicate that RelA signaling in *Scgb1a1*^+^ cells is a major mediator of HDM-induced airway cellular inflammation.

To confirm RelA KD was sufficient to inhibit RelA-mediated signaling, we examined the effect of TMX treatment on *Il6*, a gene downstream of the canonical NF-κB pathway in airway epithelial cells (18). We found the 6.6 ± 3.3-fold increase in *Il6* mRNA produced by HDM challenge to corn oil-treated mice was significantly reduced to that of PBS controls by TMX treatment (1.2 ± 0.5-fold, *P* = 0.004, Fig. 2*G*). By ELISA, IL6 in the BALF was similarly induced from 0.2 to 11.8 pg/mL by HDM treatment in corn oil-treated controls and substantially inhibited to 0.2 pg/mL by TMX treatment (Fig. 2*H*). These data indicate that the magnitude of RelA KD was sufficient to deplete RelA in Scgb1a1^+^ epithelium and functionally antagonize RelA-dependent gene activity.

### *Scgb1a1*^+^ Cell Innate Signaling Mediates Expression of Epithelial-Derived Th2 Polarization and Mucous Production

**Figure 3.**
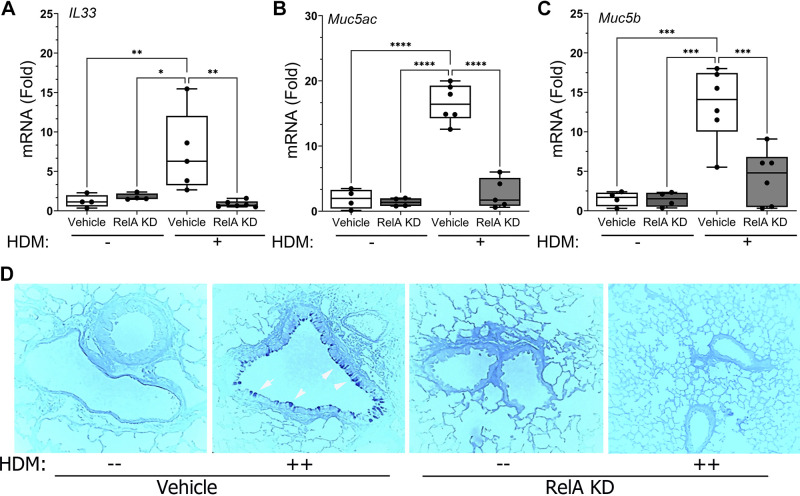
RelA knockdown (KD) in the Scgb1a1 progenitor cell blocks house dust mite (HDM) induced epithelial-derived mucus expression. *Scgb1a1*-CreER^TM^ × RelA^fl/fl^ mice (both sexes) in the C57BL/6J background were administered tamoxifen (TMX) or vehicle intraperitoneally, rested and challenged with repetitive HDM or PBS intranasally prior to total RNA extraction (Fig. 2*A*). *A*: Q-RT-PCR of *Il33* mRNA. Shown is fold change in normalized *Il33* mRNA expression over wild-type (WT) control. Symbols are individual animals (*n* = 6 samples/treatment group). Boxes are 25–75% interquartile range. *B*: effects on mucus secretion. Shown is fold change in normalized *Muc5ac* mRNA expression over WT control. *C*: fold change of normalized *Muc5b* mRNA over WT control. *D*: Alcian blue staining for mucous metaplasia. Note the dramatic increase in epithelial mucin in the vehicle-treated animals after HDM challenge (white arrowheads). Statistical symbols are: *P < 0.05; **P < 0.01; ***P < 0.005; ****P < 0.0001, post hoc Tukey.

We next assessed whether TMX affected the induction of epithelial-derived Th2 polarizing cytokines. We first examined if HDM had an effect on IL-33, a member of the IL1 family, abundantly expressed in epithelial cells and stimulator of group 2 innate lymphoid cells (34). We observed that HDM induced a robust 7.4 ± 5.1-fold increase in *Il-33* in corn oil-treated mice (*P* = 0.01 post hoc Tukey, *n* = 6; Fig. 3*A*) and this was significantly reduced to that of PBS-challenged controls at 0.9 ± 0.4-fold in the RelA KD after HDM challenge (*P* < 0.003, post hoc, *n* = 6; Fig. 3*A*).

The gel-forming mucins, MUC 5ac and -5b, are produced by mucous metaplasia in response to allergen challenge in AA. We found that HDM induced *Muc 5ac* mRNA expression by 17 ± 2-fold in control mice (*n* = 6; *P* < 0.0001, post hoc Tukey, Fig. 3*B*); this induction was reduced to 2.7 ± 2-fold in the RelA KD (*P* < 0.0001, *n* = 5; Fig. 3*B*). Similarly, the 13.4 ± 4.7-fold increase in *MUC5b* mRNA in control mice was reduced to 4.2 ± 3.5-fold in the RelA KD mice (*P* < 0.001, *n* = 6; Fig. 3*C*). To extend these findings, cellular mucins were quantitated by Alcian blue staining in the same tissues. We observed that HDM induced a dramatic increase in Alcian-blue stained epithelium that was completely inhibited by RelA KD (Fig. 3*D*). These data indicate that RelA signaling mediated Th2 cytokine production and was required for HDM-induced mucous metaplasia.

### RelA Signaling in *Scgb1a1*^+^ Cell Population Is Required for Th2 Polarization and Sensitization

**Figure 4.**
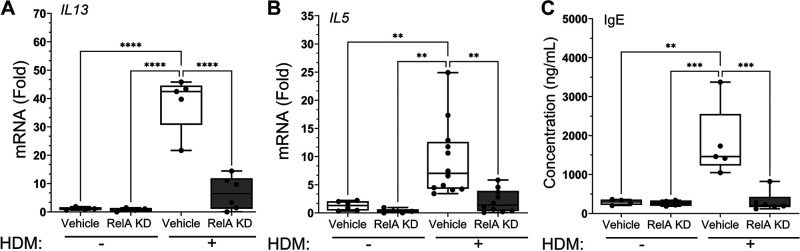
RelA signaling in the Scgb1a1 progenitor cell required for Th2 polarization and allergic sensitization. *Scgb1a1*-CreER^TM^ × RelA^fl/fl^ mice were treated with tamoxifen (TMX) or corn oil (vehicle) intraperitoneally, rested and challenged with repetitive house dust mite (HDM) or PBS intranasally prior to total RNA extraction. *A*: fold change in *Il13* mRNA. *B*: fold change in *Il5* mRNA. *C*: quantitation of IgE. Total serum IgE was measured in each group. Statistical symbols are: **P < 0.01; ***P < 0.005; ****P < 0.0001, post hoc Tukey.

The effect of RelA KD on the expression of downstream Th2 cytokines was examined next. Of these is IL13, a Th2 signature secreted by lymphocytes, eosinophils, and basophils that plays an important role in allergic sensitization producing IgE class switching. We observed HDM produced a robust 39 ± 10-fold induction of *Il13* mRNA in corn oil-treated mice; this was reduced to 6.6 ± 6-fold in the RelA KD animals (*P* < 0.0001 post hoc Tukey, *n* = 6; Fig. 4*A*). A less robust induction of *Il5* mRNA was observed, where HDM increased *Il5* mRNA 9.4 ± 6.5-fold in corn oil-treated mice; this was reduced to 2.1 ± 2-fold in the RelA KD (*P* = 0.002; post hoc, *n* = 10; Fig. 4*B*).

Finally the effect of RelA KD on IgE was determined. In corn oil-treated animals, IgE levels increased from 291 ± 71 ng/mL to 1,807 ± 907 ng/mL in response to HDM challenge (Fig. 3*G*). In the RelA KD, IgE levels were 263 ± 73 ng/mL in PBS-challenged mice (ns compared with corn oil-treated) but only increased to 306 ± 258 ng/mL after HDM challenge (*P* = 0.0005, *n* = 6; Fig. 4*C*). These data indicate that RelA signaling in *Scgb1a1*^+^ progenitor cells was required for HDM-induced Th2 polarization and allergic sensitization.

### RelA KD Prevents HDM-Induced Small Airways Resistance and AHR

**Figure 5.**
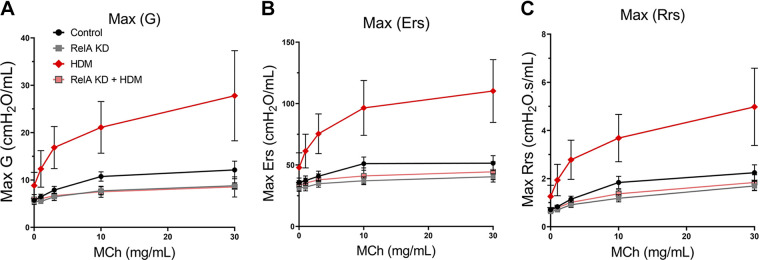
Effect of RelA knockdown (KD) on airway mechanics. *Scgb1a1*-CreER^TM^ × RelA^fl/fl^ mice were treated with tamoxifen (TMX) or corn oil intraperitoneally, rested and challenged with repetitive house dust mite (HDM) or PBS intranasally prior to measurement of lung mechanics by Flexi-Vent. *A*: effects of HDM exposure on tissue damping (G). Shown are means ± SD of *n* = 6 animals in response to increasing methacholine (MCh) administration. Note the superimposition of the RelA KD with or without HDM at levels below that of wild type (WT). *B*: effects on RelA KD on HDM-induced Elastance (Ers). Ers reflects tissue viscoelasticity and small airways resistance of the small airways. *C*: effects on RelA KD on HDM-induced Resistance (Rrs).

Using broadband forced oscillation technique measurements in response to increasing concentrations of methacholine (MCh), we sought to determine the effects of RelA KD on airway physiology in corn oil- and TMX-treated mice (35). First, we examined the effects of RelA KD on tissue dampening (*G*), as this reflects tissue viscoelasticity and small airways resistance (36). We observed that *G* was markedly affected by TMX treatment, with HDM challenge producing a threefold increase over PBS challenge in corn oil-treated mice. In contrast, RelA KD mice (either HDM- or PBS-challenged) displayed *G* values below that of control mice (Fig. 5*A*).

Not only is tissue dampening important physiological component in AA, but airway hyperreactivity can also be measured through maximum elastance (Ers) and overall resistance (Rrs) in response to MCh, thus we noted that HDM challenge induced approximately threefold increase in Ers in HDM challenged corn oil-treated mice over that of PBS challenge (Fig. 5*B*). Consistent with the effects on *G*, Rrs, a value representing both conducting and peripheral resistance (35), were also increased fourfold by HDM challenge in corn oil-treated mice. This induction was reduced to levels below control values by RelA KD, irrespective of whether they were challenged with HDM or PBS (Fig. 5*C*). Collectively, these data indicate that RelA signaling in *Scgb1a1*^+^ progenitors mediates HDM-induced changes in peripheral resistance as well as AHR.

### HDM Induces Epithelial Plasticity through RelA Signaling in the *Scgb1a1*^+^ Progenitor

**Figure 6.**
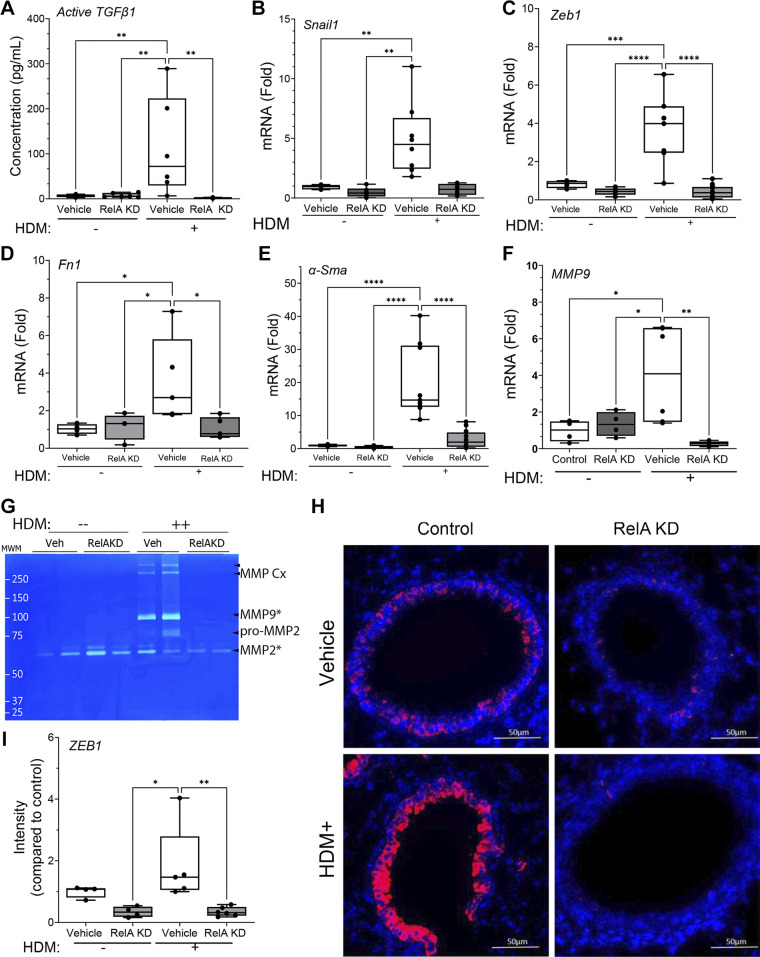
House dust mite (HDM) induces epithelial plasticity through RelA signaling and mucosal transforming growth factor (TGF)β secretion. Tamoxifen (TMX)-treated [RelA knockdown (KD)] or corn oil-treated (vehicle) *Scgb1a1*-CreER^TM^ × RelA^fl/fl^ mice were repetitively challenged with repetitive HDM or PBS intranasally. *A*: secretion of activated TGFβ. Activated TGFβ1 was measured in decellularized bronchoalveolar lavage fluid (BALF) by ELISA. Shown is active TGFβ1 concentration for each treatment group. Each symbol represents a separate animal (*n* = 5 animals/group). Activation of plasticity pathways was determined by measurement of fold change of normalized mRNA in total lung RNA or the following genes: *Snail1* (*B*); *Zeb1* (*C*); *Fn1* (*D*); *αSma/ACTA2* (*E*); and *Mmp9* (*F*) mRNA. *G*: determination of enzymatically active metalloproteinase (MMP)9. Zymogram of volume normalized BALF for vehicle (Veh) or TMX (RelA KD)-treated mice in after PBS or HDM (+) treatment. Location of precursor (pro) and active MMPs (*) are shown at right. *H*: HDM induces epithelial ZEB1 expression. Lung sections from corn oil (vehicle)- or TMX (RelA KD) treated mice were stained with anti-ZEB1 antibody (Ab) followed by secondary goat anti-Rabbit (red). Nuclei were counterstained with DAPI (blue). Immunofluorescence microscopic images were obtained. Note the intense red staining epithelium in the small bronchioles. *I*: quantitation of ZEB1 immunostaining. Fluorescence images were quantified by FIJI. Means ± SD of fluorescence intensity normalized to WT control for *n* = 4 animals/group. Statistical symbols are: *P < 0.05; **P < 0.01; ***P < 0.005; ****P < 0.0001, post hoc Tukey.

Epithelial plasticity is a highly conserved injury/repair process for repopulation of injured epithelial surfaces. Earlier we observed that RelA is a master regulator of the coordinated plasticity program in model small airway epithelial cells activated by TGFRβ or TLR3 agonists (18, 31, 33). Here, RelA functions as a direct activator of SNAI1 expression, perturbing the negative autoregulatory loop with ZEB1 and miR-34/200 enabling expression and translation of these mesenchymal transcription factors (37). To establish whether this pathway was functional in vivo, we examined whether RelA KD in *Scgb1a1*^+^ epithelial cells affected plasticity signatures. We first examined whether HDM-induced mucosal TGFβ1 production was RelA dependent. We observed that HDM significantly induced 75 pg/mL of active TGFβ1 in vehicle-treated mice that was significantly inhibited to less than PBS challenged mice (Fig. 6*A*, *P* < 0.01, post hoc). These data confirm prior observations that HDM activates mucosal TGFβ1 signaling (38), and demonstrates this pathway is RelA dependent.

We next examined whether HDM activated plasticity. In corn oil-treated mice, repetitive HDM induced a 4.9 ± 3.0-fold increase in *Snai1* mRNA, whereas RelA KD mice only expressed *Snail1* to 0.73 ± 0.4-fold (*P* = 0.002, post hoc Tukey, *n* = 8; Fig. 6*B*), a level less than that of untreated controls. In a similar pattern, HDM increased *Zeb1* mRNA by 3.7 ± 1.7-fold, *Fn1* mRNA by 2.2 ±-fold, *αSma*/*Acta2* by 15 ± -fold, and *Mmp9* mRNAs by 4.2 ± 2.6-fold in corn oil-treated mice (Fig. 6, *B–F*). These plasticity markers were reduced to levels less than that of untreated controls in the RelA KD mice (Fig. 6, *B–F*). Noting that IL6 is also a component of TGFβ-induced plasticity network (18), these data indicate that HDM activates mucosal plasticity in a RelA-dependent manner.

Extracellular MMP activity plays a major role in epithelial plasticity through extracellular matrix (ECM) remodeling as well as direct signaling actions on tissue resident lung cells and is tightly regulated by tissue inhibitors. To confirm that the enhanced MMP9 expression indicated changes in enzymatically active MMP activity, we measured BALF MMP activity using in-gel zymography. Here, we observed that HDM induced the production of active MMP9 and that its activation was lost in the RelA KD mice (Fig. 6*G*).

To confirm that HDM induced expression of epithelial plasticity markers, lung sections from corn oil-treated or RelA KD mice were stained with anti-ZEB1 Abs. In corn oil-treated mice challenged with PBS, a sparse staining pattern of ZEB1 was observed in the small bronchioles (Fig. 6*D*). By contrast, HDM challenge increased ZEB1 staining primarily in the epithelium (Fig. 6*D*). In TMX-treated mice, ZEB1 staining in both PBS and HDM challenges (Fig. 6*D*). After quantitation, the 1.8 ± 0.4 arbitrary fluorescence units (AFUs) observed in HDM-treated WT mice was significantly reduced to 0.4 ± 0.2 AFUs in either PBS- or HDM-challenged RelA KD mice (Fig. 6*E*). These data indicate that HDM activates epithelial plasticity in a RelA-dependent manner in vivo.

### HDM Disrupts the Epithelial Permeability Barrier

**Figure 7.**
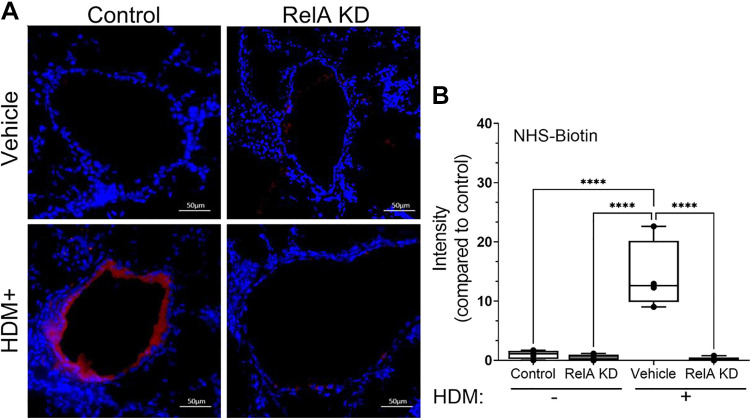
House dust mite (HDM) disrupts epithelial barrier dysfunction through RelA signaling. *Scgb1a1*-CreER^TM^ × RelA^fl/fl^ mice were treated with corn oil or tamoxifen (TMX) and repetitively challenged with repetitive HDM or PBS intranasally. Prior to euthanasia, mice were administered NHS-Biotin intranasally. *A*: lung sections were fixed and stained with streptavidin (red) and counter stained with DAPI (blue). *B*: quantitation of interstitial NHS-Biotin. Fluorescence images were quantified by FIJI. Means ± SD of fluorescence intensity normalized to wild-type (WT) control for *n* = 4 animals/group. ****P < 0.0001.

We were intrigued by the finding that repetitive HDM administration depleted cell-surface ZO-1, an important component of epithelial tight junctions important in restricting macromolecular penetration into the interstitium (Fig. 1*D*). However, the physiological significance of this finding was unclear. To directly measure the impact of HDM treatment on epithelial permeability, corn oil-treated or RelA KD mice were subjected to an NHS-Biotin permeability assay. NHS-Biotin is a membrane impermeable probe that does not cross intact epithelial barriers; however with tight junction disruption, NHS-Biotin is able to penetrate paracellular spaces that can be visualized with Texas red-streptavidin staining (23). In corn oil-treated mice challenged with PBS, no evidence of NHS-Biotin permeability is seen. In distinct contrast, NHS-Biotin staining is seen in the airway mucosa after repetitive HDM challenge; no NHS Biotin permeabilization is seen in the RelA KD mice (Fig. 7*A*). After quantitation of biological replicates, we observe that HDM induces a 14.2 ± 5.0-fold increase in NHS permeability in corn oil-treated mice that is reduced to 0.19 ± 0.3-fold in the RelA KD mice (Fig. 7*B*; *P* < 0.0001, post hoc Tukey). These data indicate that RelA signaling and epithelial plasticity are associated with disruption of the epithelial permeability barrier in the small airways.

### MMP2/9 Mediates HDM-Induced Epithelial Permeability Barrier Disruption

We noted the effect of HDM on *Mmp9* expression was RelA dependent (Fig. 6*F*), which is consistent with our earlier systems-level work in small airway cell plasticity that found that RelA drives expression of matrix metalloproteinases in response to TGFβ ligands (18), TLR3 agonists (33), and respiratory virus infection (30). In particular, MMP9 is a multifunctional effector of the epithelial injury-response pathway, responsible for ECM remodeling through its gelatinase activity, cleavage of cell-surface CDH1, and directly activating subepithelial myofibroblast transition (30), all characteristics of remodeling in AA. We therefore hypothesized that MMP secretion may play an important role in HDM-induced barrier dysfunction. To test this, we measured the effect of HDM on MMP expression in wild-type (WT) C57BL/6 mice and examined the effect of a potent and competitive MMP2/9 inhibitor (MMPi; SB-3CT) on HDM-induced permeability. SB-3CT inhibits MMP-9 with a *K*_i_ of ∼14 nM with a lower affinity for MMP-2 of 600 nM (39).

**Figure 8.**
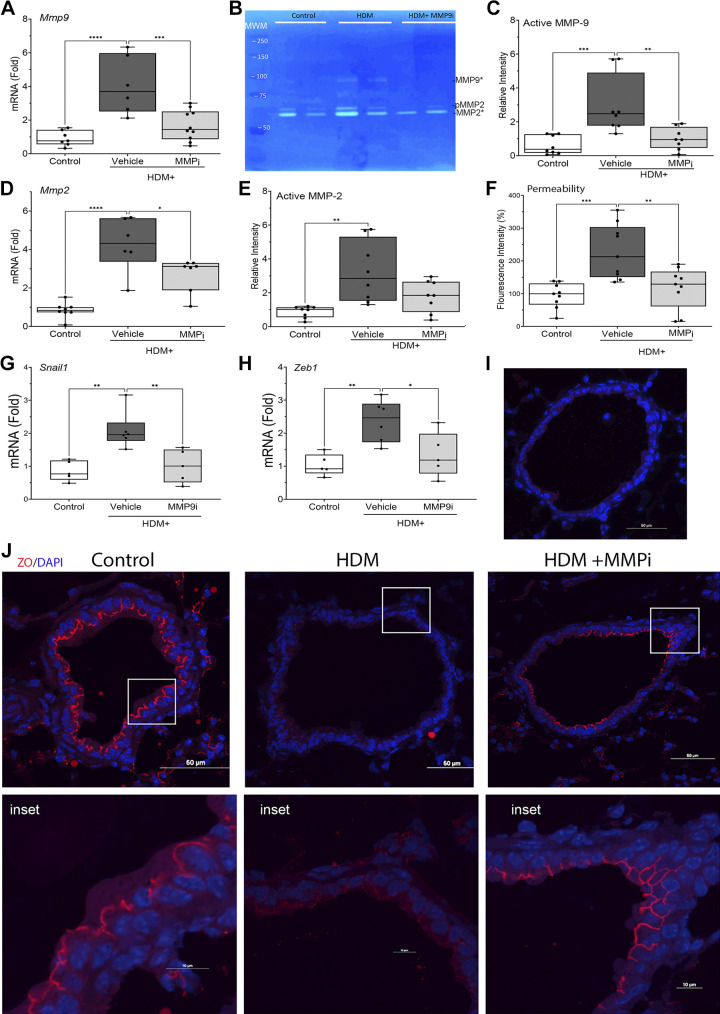
Role of metalloproteinase (MMP)2/9 in house dust mite (HDM)-induced barrier disruption. Wild-type C57BL/6 mice (*n* = 8 animals/group, mixed sexes) were repetitively challenged with PBS (Control), or HDM ± metalloproteinase inhibitor (MMPi) (SB-3CT via the IP route). *A*: fold change in normalized *Mmp9* mRNA. *B*: MMP activity in bronchoalveolar lavage fluid (BALF). Shown is a representative gel of a zymogram from the treatment groups. MMP9*, enzymatically active MMP9, pMMP2, pro-MMP2; MMP2* active MMP2. Molecular weight markers (MWM, in kDa) are shown at left. *C*: enzymatically active MMP9 in BALF. Quantitation of zymography; each symbol is a separate animal. *D*: fold change in normalized *Mmp2* mRNA. *E*: MMP2 enzymatic activity by zymogram. *F*: MMP9i blocks HDM-induced epithelial permeability. Fluorescence intensity of FITC-Dextran measured in plasma. *G* and *H*: HDM-induced epithelial plasticity is driven by MMP. *G*: fold change in normalized *Snai1* mRNA. *H*: fold change in Zeb1 mRNA.  *I* and *J*: immunofluorescence microscopy (IFM) of ZO-1 in small airways. *I*: staining produced by IgG (red). Nuclei are stained with DAPI (Blue). Scale bar, 50 µm. *J*: anti-zona occludens (ZO)-1 staining (red). *Inset*, zoomed images of regions outlined in white box, above. Note the reduced ZO-1 staining in the HDM-treated animals and disruption of the architecture. Statistical symbols are: *P < 0.05, **P < 0.01; ***P < 0.005; ****P < 0.0001, post hoc Tukey.

To confirm that HDM induces MMP9 expression, *Mmp9* mRNA was measured in total lung RNA in control versus vehicle- or MMPi-treated WT mice challenged with HDM. In vehicle-treated controls, HDM increased *Mmp9* mRNA by 4 ± 1.7-fold (*P* < 0.001; *n* = 6; Fig. 8*A*) that was reduced to 1.76 ± 0.9-fold with MMPi administration (*P* < 0.001; *n* = 10; Fig. 8*A*). MMP9 enzymatic activity is tightly regulated by the presence of tissue metalloproteinase inhibitors. To directly measure extracellular MMP9 activity, in-gel zymography of BALF was performed. Here, we observed that HDM increased MMP9 gelatinase activity from 0.62 ± 0.5-fold to 3.0 ± 1.7-fold in vehicle-treated mice that was reduced to 1.0 0.65-fold by MMPi treatment 4.4 ± 2.5-fold relative to vehicle-treated animals (*P* < 0.01, *n* = 8; Fig. 8, *B* and *C*).

Similarly, we noted *Mmp-2* mRNA was induced 4.3 ± 1.4-fold by HDM challenge (*P* < 0.0001; *n* = 6; Fig. 8*D*) and was reduced by MMPi. MMP2 enzymatic activity was increased 3.3 ± 1.8-fold in the HDM-treated animals and was reduced to untreated control levels by MMPi (Fig. 8*E*).

As an independent measurement of epithelial permeability, we measured permeability to FITC-Dextran. These data confirmed that HDM induced twofold increase in permeability versus controls (*P* < 0.001, Fig. 8*F*) that was also normalized by MMPi treatment. These data confirm that MMP-2 and -9 are induced by HDM at the gene expression and enzymatic level and whose activity mediates HDM-induced permeability.

### MMPi Reduces HDM-Induced Epithelial Plasticity

Systems level studies have shown that epithelial plasticity is maintained by transcription factor cliques under autoregulatory control from epithelial growth factors (18, 40). Importantly, the TGFβ autocrine loop is dependent on tissue metalloproteinases that release latent TGFβ from its matricellular stores. We sought to determine whether the MMPi affected this autocrine regulatory loop by examining the effect on the Snail1-Zeb1 core mesenchymal transcription factors. Consistent with our earlier findings, HDM induced a 2.0 ± 0.6-fold induction of *Snail1* mRNA expression that was significantly reduced to 1 ± 0.5-fold by MMPi treatment (*P* < 0.01, Fig. 8*G*). A similar significant inhibitory effect was observed for Zeb1 expression (*P* < 0.05, Fig. 8*H*). Collectively, these data indicate that MMPs are produced by HDM and participate in the maintenance of cellular plasticity.

### HDM Disrupts Cell Surface Zona Occludens-1-Containing Adherens Junctions via MMP2/9

Extending our earlier findings that HDM disrupts cell-surface ZO-1 adherens junctions (Fig. 1*E*), we conducted IFM on tissue sections from the MMPi experiment. In control mice, we observed a characteristic continuous apical staining of ZO-1 in the small airways (Fig. 8*J*). By contrast, HDM treatment resulted in a reduction of total ZO-1 staining, disruption of the uniform apical continuity, and cytoplasmic re-distribution (Fig. 8*J*). In striking contrast, the apical ZO-1 staining was preserved in MMPi-treated animals (Fig. 8*J*). These data indicate that MMP2/9 enzymatic activity mediates HDM-induced epithelial barrier dysfunction, at least in part, through dissolution of ZO-1-containing adherens junctions.

## DISCUSSION

The respiratory epithelium provides a physical, functional, and immunologic barrier to protect the host from allergens and pathogens. Although epithelial barrier disruption is one of the earliest abnormalities in AA (41, 42), the mechanisms on how disruption occurs and its effects on sensitization and Th2 polarization are not fully understood. In this study, we examine how innate signaling is related to epithelial plasticity, barrier function, and Th2 polarization in response to the ubiquitous, perennial indoor HDM allergen (32) representing the major target of IgE in sensitized individuals (7). We find that HDM acutely activates RelA signaling via the canonical pathway, and induces epithelial plasticity in EpCAM+ cells. In addition, HDM induces a functional disruption of the epithelial permeability barrier and is blocked by RelA depletion in *Scgb1a1*^+^ progenitor cells. Importantly, repetitive HDM exposure activates epithelial IL13 production, mucous metaplasia, downstream Th2 polarization, and IgE formation in a manner dependent on epithelial RelA. Linking these changes to physiological effects, we observe that RelA signaling not only mediates lower airway viscoelastic resistance, but also mediates AHR. Our data further show that epithelial RelA-dependent plasticity activates expression and enzymatic activity of MMP2/9 that induce barrier dysfunction through a mechanism involving disruption of ZO-1 containing adherens junctions. These data establish that the *Scgb1a1*^+^ progenitor cell innate response to HDM is required for epithelial plasticity and airway barrier disruption leading to allergen sensitization and AHR.

Club cells derived from *Scgb1a1^+^* precursors are one of five major epithelial cell types identified by single-cell sequencing and cell lineage studies (22, 27). Lineage inference suggests that *Scgb1a1^+^* precursors differentiate into Club cells, but also form goblet and multiciliated cells, enriched in the distal airway (22, 27). Building evidence suggests that this lineage plays major roles in injury/repair in the small airways/terminal bronchioles (43). To advance the understanding of this population, lung clearing coupled with immunofluorescence light sheet microscopic imaging of the native three-dimensional distribution of these cells found that these cells are distributed in the mouse lung throughout the small airways and terminal bronchioles. This localization allows these cells to function as innate sentinel cells triggering inflammation, repair, and remodeling when allergens penetrate deeply into the distal airways. Our findings extend this work to demonstrate that RelA signaling mediates allergen-induced mucous metaplasia.

Epithelial plasticity is a multistep cell-state de-differentiation program activated by epithelial growth factors in response to mucosal injury. Plasticity is driven by coordinate waves of gene expression networks under control of “cliques” of master transcription factors, including NF-κB/RelA (40). Partial epithelial mesenchymal transition enables complex phenotypic changes including dissolution of cellular tight junctions, loss of apical polarity, and expression of contractile proteins that enable cellular migration to repopulate areas of injury. Plasticity is reversible, enabling the migratory cell population to differentiate back to mature alveolar type 1 or bronchiolar epithelial cells (the so-called mesenchymal-epithelial transition). Recent systems-level interrogations by our group have shown that RelA is a master upstream regulator of viral-TLR3 and TGFβ-induced plasticity (18, 30, 33). We found that RelA mediates the transition from SMAD3 signaling to a stable expression of the SNAI1-ZEB mesenchymal regulators by disrupting its ambient negative feedback loop. Consistent with our earlier studies of TLR3 and TGFβ-induced epithelial plasticity, the genetic signature of plasticity can be induced after a single treatment, but stable accumulation of mesenchymal proteins requires repetitive stimuli (31, 44).

Repetitive activation of the RelA pathway induces SNAI1 expression to levels that enable ZEB and SNAI1 to escape miR-200 translational inhibition (45), advancing to a stable mesenchymal state. In addition, our systems-level studies integrating RNA seq with ATAC-seq have shown that the plasticity program also activates MMP and growth factor expression through a mechanism involving chromatin opening (30, 46). This chromatin remodeling is mediated by RelA recruitment of BRD4-dependent chromatin remodeling factors to phosphorylate inactive RNA polymerase II, enabling persistent MMP and TGFβ expression (16, 18). Although these molecular details are understood in some detail, the effects of plasticity in airway remodeling are still underexplored.

This study extends our understanding of plasticity in AA by demonstrating that HDM-induced RelA upregulates *Snail1* and *Zeb1* signatures in the small airways epithelium. Our findings are consistent with activation of the gene expression pathway, yet stable accumulation of SNAIL1 and ZEB1 is not observed after a single HDM stimulation, yet requires multiple exposures. These findings suggest that complex post-transcriptional regulation, such as translational regulation by miRNAs or protein degradation is also playing a role. Despite the presence of molecular mesenchymal signatures, there is no evidence that epithelium reaches a stable mesenchymal state in vivo (47). To this end, in a stable mesenchymal state, SNAI/ZEB transcriptional repressors silence ZO-1 expression at the mRNA level. Our data suggest that HDM induces only a partial EMT, because ZO-1 mRNA expression is maintained despite the presence of ZEB1.

MMPs 2 and -9 are highly related Zn-dependent endopeptidases that play a major role in inflammation (48) and fibrosis in AA (49). Of relevance to this work, studies using epithelial monolayers in vitro found that apical MMP9 induces transepithelial conductance, loss of cell-surface adherens junctions permitting viral access to the subepithelial space (50). In established AA, MMP production increases in response to allergen challenge and is produced by infiltrating leukocytes/eosinophils. Our study suggests that epithelial production of MMPs may play an important role in allergic sensitization in the naïve animal by permitting allergen to access the interstitial space.

We note that the MMPi treatment reduces expression of epithelial plasticity and MMP enzymatic activity acts to propagate epithelial plasticity. This complex interaction may be explained by the findings that MMPs are involved in processing of matricellular chemokines (49), including the release of latent TGFβ (51). Of relevance, earlier work in aeroallergen exposure elucidated that mucosal allergen exposure induces a TGFβ autocrine loop important in allergic sensitization (16, 38).

A dramatic finding from this study is the disruption of intracellular ZO-1 organization by HDM and its inhibition by MMPi. Although it is tempting to speculate that ZO-1 is directly cleaved by MMPs, little, if any, ZO-1 is exposed on the cell surface. We interpret this to mean that disruption of ZO-1 adherence junctions may be indirectly mediated by MMP cleavage of other structural components of the adherens junctions. For example it is well-demonstrated that MMPs cleave occludin (11, 52); occludin could be a direct target whose proteolysis disrupts the integrity of adherens junctions. More work will be required to understand this mechanism. We also note that dissolution of ZO-1 containing adherens junctions may have important impacts on cellular physiology other than affecting epithelial permeability. For example, in cancer-associated EMT, others have found that ZO-1 disengages from tight junctions to shuttle into the cytoplasm and nucleus where it may potentiate metalloproteinase expression (53). In addition we note that that treatment with MMPi reduces gene expression of both *MMP-2* and *-9*. This finding suggests that expressions of these two metalloproteinases are under positive feedback control, perhaps as a result of this ZO-1 signaling. The role of cytoplasmic ZO-1 on metalloproteinase expression, if any, in AA will require further investigation.

Previous studies examining the effect of allergens, pathogens, and pollutants on epithelial barrier function have suggested that the endogenous cysteine-directed proteases (Der p1) contained within the complex HDM allergen (54, 55) activate the protease activated receptor (PAR)-2 (56) and disrupt tight junctions (57) facilitating transepithelial allergen delivery (58). Our study challenges the role of the Der p1 protease, and instead suggests that endogenous MMP expression mediates plasticity, barrier disruption, sensitization, and AHR.

In summary, we report the discovery that innate signaling in *Scgb1a1^+^* precursors mediates the complex cellular responses to HDM relevant to AA. This *Scgb1a1^+^* population are progenitors of small airway that function as epithelial “sensors” of the presence of small airways control epithelial plasticity, barrier disruption, Th2 polarization, and AHR. The activation of MMPs by RelA in these specialized progenitor cells account, in large part, for the disruption of mucosal barrier in mucosal remodeling in AA. We anticipate this work will stimulate development of therapeutics for maintaining epithelial barrier.

## DATA AVAILABILITY

Data will be made available upon reasonable request.

## SUPPLEMENTAL MATERIAL

10.6084/m9.figshare.25563783 Supplemental Fig. S1 and Supplemental Table S1: https://doi.org/10.6084/m9.figshare.25563783.

## GRANTS

This work was partially supported by NIH Grants U01 AI136994 (to A.R.B.), P01 AI062885 (to A.R.B), and National Center for Advancing Translational Sciences (NCATS) UL1TR002373 (to A.R.B.).

## DISCLAIMERS

The funders had no role in the design of the study; in the collection, analyses, or interpretation of data; in the writing of the manuscript, or in the decision to publish the results.

## DISCLOSURES

No conflicts of interest, financial or otherwise, are declared by the authors.

## AUTHOR CONTRIBUTIONS

M.E.S. and A.R.B. conceived and designed research; performed experiments; analyzed data; interpreted results of experiments; prepared figures; drafted manuscript; edited and revised manuscript; and approved final version of manuscript.

## References

[1] Busse WW, Lemanske RF Jr. Asthma. N Engl J Med 344: 350–362, 2001. doi:10.1056/NEJM200102013440507. 11172168

[2] Davies DE. The role of the epithelium in airway remodeling in asthma. Proc Am Thorac Soc 6: 678–682, 2009. doi:10.1513/pats.200907-067DP. 20008875 PMC2797070

[3] Global Asthma Network. The Global Asthma Report 2014. Auckland, New Zealand: Global Asthma Network, 2014, vol. 769, p. 28–36.

[4] Wise SK, Damask C, Roland LT, Ebert C, Levy JM, Lin S, , et al International consensus statement on allergy and rhinology: allergic rhinitis—2023. Int Forum Allergy Rhinol 13: 293–859, 2014. doi:10.1002/alr.23090. 36878860

[5] Biagtan M, Viswanathan R, Bush RK. Immunotherapy for house dust mite sensitivity: where are the knowledge gaps? Curr Allergy Asthma Rep 14: 482, 2014. doi:10.1007/s11882-014-0482-0. 25354663 PMC5034865

[6] Calderón MA, Linneberg A, Kleine-Tebbe J, De Blay F, Hernandez Fernandez de Rojas D, Virchow JC, Demoly P. Respiratory allergy caused by house dust mites: what do we really know? J Allergy Clin Immunol 136: 38–48, 2015. doi:10.1016/j.jaci.2014.10.012. 25457152

[7] Bessot JC, Pauli G. [House dust mites allergens]. Rev Mal Respir 28: 475–495, 2011. doi:10.1016/j.rmr.2011.02.006. 21549903

[8] Mazurek J, Syamlal G. Prevalence of asthma, asthma attacks, and emergency department visits for asthma among working adults—National Health Interview Survey. MMWR Morb Mortal Wkly Rep 67: 377–386, 2018. doi:10.15585/mmwr.mm6713a1. 29621204 PMC5889242

[9] Gregory LG, Lloyd CM. Orchestrating house dust mite-associated allergy in the lung. Trends Immunol 32: 402–411, 2011. doi:10.1016/j.it.2011.06.006. 21783420 PMC3381841

[10] Hellings PW, Steelant B. Epithelial barriers in allergy and asthma. J Allergy Clin Immunol 145: 1499–1509, 2020. doi:10.1016/j.jaci.2020.04.010. 32507228 PMC7270816

[11] Steelant B, Farré R, Wawrzyniak P, Belmans J, Dekimpe E, Vanheel H, Van Gerven L, Kortekaas Krohn I, Bullens DMA, Ceuppens JL, Akdis CA, Boeckxstaens G, Seys SF, Hellings PW. Impaired barrier function in patients with house dust mite-induced allergic rhinitis is accompanied by decreased occludin and zonula occludens-1 expression. J Allergy Clin Immunol 137: 1043–1053.e5, 2016. doi:10.1016/j.jaci.2015.10.050. 26846377

[12] Hammad H, Plantinga M, Deswarte K, Pouliot P, Willart MA, Kool M, Muskens F, Lambrecht BN. Inflammatory dendritic cells—not basophils—are necessary and sufficient for induction of Th2 immunity to inhaled house dust mite allergen. J Exp Med 207: 2097–2111, 2010. doi:10.1084/jem.20101563. 20819925 PMC2947072

[13] Lambrecht BN, Hammad H. Allergens and the airway epithelium response: gateway to allergic sensitization. J Allergy Clin Immunol 134: 499–507, 2014. doi:10.1016/j.jaci.2014.06.036. 25171864

[14] Hosoki K, Boldogh I, Aguilera-Aguirre L, Sun Q, Itazawa T, Hazra T, Brasier AR, Kurosky A, Sur S. Myeloid differentiation protein 2 facilitates pollen- and cat dander-induced innate and allergic airway inflammation. J Allergy Clin Immunol 137: 1506–1513.e2, 2016. doi:10.1016/j.jaci.2015.09.036. 26586036 PMC4860180

[15] Hosoki K, Redding D, Itazawa T, Chakraborty A, Tapryal N, Qian S, Qi H, Aguilera-Aguirre L, Brasier AR, Phani VS, Hazra TK, Boldogh I, Sur S. Innate mechanism of pollen- and cat dander-induced oxidative stress and DNA damage in the airways. J Allergy Clin Immunol 140: 1436–1439.e5, 2017. doi:10.1016/j.jaci.2017.04.044. 28583369

[16] Skibba ME, Xu X, Weiss K, Huisken J, Brasier AR. Role of Secretoglobin(+) (club cell) NFκB/RelA-TGFβ signaling in aero-allergen-induced epithelial plasticity and subepithelial myofibroblast transdifferentiation. Respir Res 22: 315, 2021. doi:10.1186/s12931-021-01910-w. 34930252 PMC8690490

[17] Tully JE, Hoffman SM, Lahue KG, Nolin JD, Anathy V, Lundblad LKA, Daphtary N, Aliyeva M, Black KE, Dixon AE, Poynter ME, Irvin CG, Janssen-Heininger YMW. Epithelial NF-κB orchestrates house dust mite-induced airway inflammation, hyperresponsiveness, and fibrotic remodeling. J Immunol 191: 5811–5821, 2013. doi:10.4049/jimmunol.1301329. 24227776 PMC3858534

[18] Tian B, Widen SG, Yang J, Wood TG, Kudlicki A, Zhao Y, Brasier AR. The NF-κB subunit RELA is a master transcriptional regulator of the committed epithelial-mesenchymal transition in airway epithelial cells. J Biol Chem 293: 16528–16545, 2018. doi:10.1074/jbc.RA118.003662. 30166344 PMC6200927

[19] Tian B, Hosoki K, Liu Z, Yang J, Zhao Y, Sun H, Zhou J, Rytting E, Kaphalia L, Calhoun WJ, Sur S, Brasier AR. Mucosal bromodomain-containing protein 4 mediates aeroallergen-induced inflammation and remodeling. J Allergy Clin Immunol 143: 1380–1394.e9, 2019. doi:10.1016/j.jaci.2018.09.029. 30321559 PMC6490683

[20] Zhao Y, Jamaluddin M, Zhang Y, Sun H, Ivanciuc T, Garofalo RP, Brasier AR. Systematic analysis of cell-type differences in the epithelial secretome reveals insights into the pathogenesis of respiratory syncytial virus-induced lower respiratory tract infections. J Immunol 198: 3345–3364, 2017. doi:10.4049/jimmunol.1601291. 28258195 PMC5380581

[21] Brasier AR. Mechanisms how mucosal innate immunity affects progression of allergic airway disease. Expert Rev Respir Med 13: 349–356, 2019. doi:10.1080/17476348.2019.1578211. 30712413 PMC6510479

[22] Zaragosi L, Deprez M, Barbry P. Using single-cell RNA sequencing to unravel cell lineage relationships in the respiratory tract. Biochem Soc Trans 48: 327–336, 2020. doi:10.1042/BST20191010. 31922198

[23] Wiesner DL, Merkhofer RM, Ober C, Kujoth GC, Niu M, Keller NP, Gern JE, Brockman-Schneider RA, Evans MD, Jackson DJ, Warner T, Jarjour NN, Esnault SJ, Feldman MB, Freeman M, Mou H, Vyas JM, Klein BS. Club cell TRPV4 serves as a damage sensor driving lung allergic inflammation. Cell Host Microbe 27: 614–628.e6, 2020. doi:10.1016/j.chom.2020.02.006. 32130954 PMC7305569

[24] Tian B, Yang J, Zhao Y, Ivanciuc T, Sun H, Wakamiya M, Garofalo RP, Brasier AR. Central role of the NF-κB pathway in the Scgb1a1-expressing epithelium in mediating respiratory syncytial virus-induced airway inflammation. J Virol 92: e00441-18, 2018. doi:10.1128/JVI.00441-18. 29593031 PMC5952137

[25] Tian B, Liu Z, Yang J, Sun H, Zhao Y, Wakamiya M, Chen H, Rytting E, Zhou J, Brasier AR. Selective antagonists of the bronchiolar epithelial NF-κB-bromodomain-containing protein 4 pathway in viral-induced airway inflammation. Cell Rep 23: 1138–1151, 2018. doi:10.1016/j.celrep.2018.03.106. 29694891 PMC6020052

[26] Liu Q, Liu K, Cui G, Huang X, Yao S, Guo W, Qin Z, Li Y, Yang R, Pu W, Zhang L, He L, Zhao H, Yu W, Tang M, Tian X, Cai D, Nie Y, Hu S, Ren T, Qiao Z, Huang H, Zeng YA, Jing N, Peng G, Ji H, Zhou B. Lung regeneration by multipotent stem cells residing at the bronchioalveolar-duct junction. Nat Genet 51: 728–738, 2019. [Erratum in Nat Genet 51: 766, 2019]. doi:10.1038/s41588-019-0346-6. 30778223

[27] Deprez M, Zaragosi L-E, Truchi M, Becavin C, Ruiz García S, Arguel M-J, Plaisant M, Magnone V, Lebrigand K, Abelanet S, Brau F, Paquet A, Pe'er D, Marquette C-H, Leroy S, Barbry P. A single-cell atlas of the human healthy airways. Am J Respir Crit Care Med 202: 1636–1645, 2020. doi:10.1164/rccm.201911-2199OC. 32726565

[28] Avila PC. Plasticity of airway epithelial cells. J Allergy Clin Immunol 128: 1225–1226, 2011. doi:10.1016/j.jaci.2011.10.006. 22133319 PMC3229749

[29] Aiello NM, Maddipati R, Norgard RJ, Balli D, Li J, Yuan S, Yamazoe T, Black T, Sahmoud A, Furth EE, Bar-Sagi D, Stanger BZ. EMT subtype influences epithelial plasticity and mode of cell migration. Dev Cell 45: 681–695.e4, 2018. doi:10.1016/j.devcel.2018.05.027. 29920274 PMC6014628

[30] Xu X, Qiao D, Dong C, Mann M, Garofalo RP, Keles S, Brasier AR. The SWI/SNF-related, matrix associated, actin-dependent regulator of chromatin A4 core complex represses respiratory syncytial virus-induced syncytia formation and subepithelial myofibroblast transition. Front Immunol 12: 633654, 2021. doi:10.3389/fimmu.2021.633654. 33732255 PMC7957062

[31] Tian B, Patrikeev I, Ochoa L, Vargas G, Belanger KK, Litvinov J, Boldogh I, Ameredes BT, Motamedi M, Brasier AR. NF-κB mediates mesenchymal transition, remodeling, and pulmonary fibrosis in response to chronic inflammation by viral RNA patterns. Am J Respir Cell Mol Biol 56: 506–520, 2017. doi:10.1165/rcmb.2016-0259OC. 27911568 PMC5449514

[32] Arlian LG, Morgan MS, Neal JS. Dust mite allergens: ecology and distribution. Curr Allergy Asthma Rep 2: 401–411, 2002. doi:10.1007/s11882-002-0074-2. 12165207

[33] Tian B, Liu Z, Litvinov J, Maroto R, Jamaluddin M, Rytting E, Patrikeev I, Ochoa L, Vargas G, Motamedi M, Ameredes BT, Zhou J, Brasier AR. Efficacy of novel highly specific bromodomain-containing protein 4 inhibitors in innate inflammation-driven airway remodeling. Am J Respir Cell Mol Biol 60: 68–83, 2019. doi:10.1165/rcmb.2017-0445OC. 30153047 PMC6348724

[34] Liew FY, Girard J-P, Turnquist HR. Interleukin-33 in health and disease. Nat Rev Immunol 16: 676–689, 2016. doi:10.1038/nri.2016.95. 27640624

[35] McGovern TK, Robichaud A, Fereydoonzad L, Schuessler TF, Jg M. Evaluation of respiratory system mechanics in mice using the forced oscillation technique. J Vis Exp e50172, 2013. doi:10.3791/50172. 23711876 PMC3684007

[36] Siddiqui S, Tsuchiya K, Risse PA, Bullimore SR, Benedetti A, Martin JG. Site of allergic airway narrowing and the influence of exogenous surfactant in the Brown Norway rat. PLoS One 7: e29381, 2012. doi:10.1371/journal.pone.0029381. 22276110 PMC3261862

[37] Tian B, Zhao Y, Sun H, Zhang Y, Yang J, Brasier AR. BRD4 mediates NF-kappaB-dependent epithelial-mesenchymal transition and pulmonary fibrosis via transcriptional elongation. Am J Physiol Lung Cell Mol Physiol 311: L1183–L1201, 2016. doi:10.1152/ajplung.00224.2016. 27793799 PMC5206405

[38] Denney L, Byrne Adam J, Shea Thomas J, Buckley James S, Pease James E, Herledan GMF, Walker Simone A, Gregory Lisa G, Lloyd Clare M. Pulmonary epithelial cell-derived cytokine TGF-β1 is a critical cofactor for enhanced innate lymphoid cell function. Immunity 43: 945–958, 2015. doi:10.1016/j.immuni.2015.10.012. 26588780 PMC4658339

[39] Lee M, Chen Z, Tomlinson BN, Gooyit M, Hesek D, Juárez MR, Nizam R, Boggess B, Lastochkin E, Schroeder VA, Wolter WR, Suckow MA, Cui J, Mobashery S, Gu Z, Chang M. Water-soluble MMP-9 inhibitor reduces lesion volume after severe traumatic brain injury. ACS Chem Neurosci 6: 1658–1664, 2015. doi:10.1021/acschemneuro.5b00140. 26241578 PMC5800744

[40] Chang H, Liu Y, Xue M, Liu H, Du S, Zhang L, Wang P. Synergistic action of master transcription factors controls epithelial-to-mesenchymal transition. Nucleic Acids Res 44: 2514–2527, 2016. doi:10.1093/nar/gkw126. 26926107 PMC4824118

[41] Fedorov IA, Wilson SJ, Davies DE, Holgate ST. Epithelial stress and structural remodelling in childhood asthma. Thorax 60: 389–394, 2005. doi:10.1136/thx.2004.030262. 15860714 PMC1758889

[42] Trivedi M, Denton E. Asthma in children and adults—what are the differences and what can they tell us about asthma? Front Pediatr 7: 256, 2019. doi:10.3389/fped.2019.00256. 31294006 PMC6603154

[43] Rawlins EL, Okubo T, Xue Y, Brass DM, Auten RL, Hasegawa H, Wang F, Hogan BL. The role of Scgb1a1+ Clara cells in the long-term maintenance and repair of lung airway, but not alveolar, epithelium. Cell Stem Cell 4: 525–534, 2009. doi:10.1016/j.stem.2009.04.002. 19497281 PMC2730729

[44] Tian B, Li X, Kalita M, Widen SG, Yang J, Bhavnani SK, Dang B, Kudlicki A, Sinha M, Kong F, Wood TG, Luxon BA, Brasier AR. Analysis of the TGFβ-induced program in primary airway epithelial cells shows essential role of NF-κB/RelA signaling network in type II epithelial mesenchymal transition. BMC Genomics 16: 529, 2015. doi:10.1186/s12864-015-1707-x. 26187636 PMC4506436

[45] Lu M, Jolly MK, Levine H, Onuchic JN, Ben-Jacob E. MicroRNA-based regulation of epithelial–hybrid–mesenchymal fate determination. Proc Natl Acad Sci USA 110: 18144–18149, 2013. doi:10.1073/pnas.1318192110. 24154725 PMC3831488

[46] Xu X, Qiao D, Mann M, Garofalo RP, Brasier AR. Respiratory syncytial virus infection induces chromatin remodeling to activate growth factor and extracellular matrix secretion pathways. Viruses 12: 804, 2020. doi:10.3390/v12080804. 32722537 PMC7472097

[47] Rock JR, Barkauskas CE, Cronce MJ, Xue Y, Harris JR, Liang J, Noble PW, Hogan BL. Multiple stromal populations contribute to pulmonary fibrosis without evidence for epithelial to mesenchymal transition. Proc Natl Acad Sci USA 108: E1475–E1483, 2011. doi:10.1073/pnas.1117988108. 22123957 PMC3248478

[48] Kumagai K, Ohno I, Okada S, Ohkawara Y, Suzuki K, Shinya T, Nagase H, Iwata K, Shirato K. Inhibition of matrix metalloproteinases prevents allergen-induced airway inflammation in a murine model of asthma. J Immunol 162: 4212–4219, 1999. doi:10.4049/jimmunol.162.7.4212. 10201949

[49] Parks WC, Wilson CL, López-Boado YS. Matrix metalloproteinases as modulators of inflammation and innate immunity. Nat Rev Immunol 4: 617–629, 2004. doi:10.1038/nri1418. 15286728

[50] Vermeer PD, Denker J, Estin M, Moninger TO, Keshavjee S, Karp P, Kline JN, Zabner J. MMP9 modulates tight junction integrity and cell viability in human airway epithelia. Am J Physiol Lung Cell Mol Physiol 296: L751–L762, 2009. doi:10.1152/ajplung.90578.2008. 19270179 PMC2681350

[51] Kobayashi T, Kim H, Liu X, Sugiura H, Kohyama T, Fang Q, Wen FQ, Abe S, Wang X, Atkinson JJ, Shipley JM, Senior RM, Rennard SI. Matrix metalloproteinase-9 activates TGF-β and stimulates fibroblast contraction of collagen gels. Am J Physiol Lung Cell Mol Physiol 306: L1006–L1015, 2014. doi:10.1152/ajplung.00015.2014. 24705725 PMC4042193

[52] Pan R, Liu W, Liu KJ. MMP-2/9-cleaved occludin promotes endothelia cell death in ischemic stroke. Brain Hemorrhages 2: 63–70, 2021. doi:10.1016/j.hest.2021.01.002.

[53] Polette M, Mestdagt M, Bindels S, Nawrocki-Raby B, Hunziker W, Foidart J-M, Birembaut P, Gilles C. β-Catenin and ZO-1: shuttle molecules involved in tumor invasion-associated epithelial-mesenchymal transition processes. Cells Tissues Organs 185: 61–65, 2007. doi:10.1159/000101304. 17587809

[54] Douwes J, Zuidhof A, Doekes G, van der Zee S, Wouters I, Marike Boezen H, Brunekreef B. (1→ 3)-β-d-glucan and endotoxin in house dust and peak flow variability in children. Am J Respir Crit Care Med 162: 1348–1354, 2000. doi:10.1164/ajrccm.162.4.9909118. 11029343

[55] Jacquet A. The role of the house dust mite-induced innate immunity in development of allergic response. Int Arch Allergy Immunol 155: 95–105, 2011. doi:10.1159/000320375. 21196753

[56] Asokananthan N, Graham PT, Stewart DJ, Bakker AJ, Eidne KA, Thompson PJ, Stewart GA. House dust mite allergens induce proinflammatory cytokines from respiratory epithelial cells: the cysteine protease allergen, Der p 1, activates protease-activated receptor (PAR)-2 and inactivates PAR-1. J Immunol 169: 4572–4578, 2002. doi:10.4049/jimmunol.169.8.4572. 12370395

[57] Wan H, Winton HL, Soeller C, Tovey ER, Gruenert DC, Thompson PJ, Stewart GA, Taylor GW, Garrod DR, Cannell MB, Robinson C. Der p 1 facilitates transepithelial allergen delivery by disruption of tight junctions. J Clin Invest 104: 123–133, 1999. doi:10.1172/JCI5844. 10393706 PMC408401

[58] Heijink IH, Postma DS, Noordhoek JA, Broekema M, Kapus A. House dust mite-promoted epithelial-to-mesenchymal transition in human bronchial epithelium. Am J Respir Cell Mol Biol 42: 69–79, 2010. doi:10.1165/rcmb.2008-0449OC. 19372245

